# Serum chitinase-3-like 1 protein is a useful biomarker to assess disease activity in ANCA-associated vasculitis: an observational study

**DOI:** 10.1186/s13075-021-02467-1

**Published:** 2021-03-08

**Authors:** Sung Soo Ahn, Taejun Yoon, Yong-Beom Park, Maria Prendecki, Gurjeet Bhangal, Stephen P. McAdoo, Sang-Won Lee

**Affiliations:** 1 Department of Internal Medicine, Yongin Severance Hospital, Yonsei University College of Medicine, 50-1 Yonsei-ro, Seodaemun-gu, Seoul, 03722 Republic of Korea; 2 Department of Medical Science, BK21 Plus Project, Yonsei University College of Medicine, Seoul, Republic of Korea; 3 Institute for Immunology and Immunological Diseases, Yonsei University College of Medicine, Seoul, Republic of Korea; 4 Division of Rheumatology, Department of Internal Medicine, Yonsei University College of Medicine, Seoul, Republic of Korea; 5 Centre for Inflammatory Disease, Department of Immunology and Inflammation, Imperial College London, Hammersmith Campus, Du Cane Road, London, W120NN UK

**Keywords:** Anti-neutrophil cytoplasmic antibody-associated vasculitis, YKL-40, Activity, Biomarker, Inflammation

## Abstract

**Background:**

To investigate whether serum chitinase-3-like 1 protein (YKL-40) is associated with disease activity in anti-neutrophil cytoplasmic antibody (ANCA)-associated vasculitis (AAV).

**Methods:**

ELISA was performed in serum samples from AAV patients who were enrolled in our prospective observational cohort to estimate levels of YKL-40. Birmingham vasculitis activity score (BVAS) (version 3), five factor score (FFS), and short form-36 (SF-36), as well as clinical and laboratory data were collected. Kidney expression of YKL-40 was assessed by immunohistochemical staining using renal biopsy tissues from ANCA-associated glomerulonephritis patients (AAGN). Severe AAV and FFS were defined as BVAS ≥ 12 and FFS ≥ 2, and the correlations between laboratory variables, BVAS, FFS, and SF-36 score were assessed using linear regression analysis. The optimal cut-off of serum YKL-40 for severe AAV and high FFS was calculated using the receiver operator characteristic curve analysis.

**Results:**

Of the included 60 patients, 32 (53.3%), 17 (28.3%), and 11 (18.3%) were classified as microscopic polyangiitis, granulomatosis with polyangiitis, and eosinophilic granulomatosis with polyangiitis. The median BVAS and FFS were 7.0 and 1.0, whereas the mean SF-36 physical and mental component scores were 50.5 and 58.3. Serum YKL-40 level was higher in patients with severe AAV and high FFS compared to those without (*p* = 0.007 and *p* < 0.001); multivariable linear regression analysis revealed that serum YKL-40 was independently associated with BVAS, FFS, and SF-36 scores. On kidney tissues obtained from AAGN patients, strong cytoplasmic staining of YKL-40 was found in cells present in inflammatory lesions. In addition, AAV patients had higher levels of serum YKL-40 compared to those with systemic lupus erythematosus, rheumatoid arthritis, osteoarthritis, and healthy control. The proportion of patients having severe AAV and high FFS was significantly higher in those with serum YKL-40 > 221.3 ng/mL and > 227.1 ng/mL than those without (relative risk 2.852 and 7.000). In 12 patients with serial YKL-40 testing, 11 patients (91.7%) exhibited a reduction in serum YKL-40 levels following a decrease in disease activity (*p* < 0.001).

**Conclusion:**

Our findings suggest that serum YKL-40 may be a clinically useful biomarker to assess AAV disease activity.

**Trial registration:**

Retrospectively registered.

## Background

Anti-neutrophil cytoplasmic autoantibody (ANCA)-associated vasculitis (AAV) is a rare disease characterised by necrotizing inflammation of predominantly small vessels [1]. ANCAs against myeloperoxidase (MPO-ANCA) or proteinase 3 (PR3-ANCA) are a serologic hallmark of AAV, even though they are not detected in all AAV patients [2]. AAV is classified into three disease categories: granulomatosis with polyangiitis (GPA), microscopic polyangiitis (MPA), and eosinophilic GPA (EGPA) based on clinical, laboratory, and pathological features [3]. In recent decades, the clinical outcome of AAV has significantly improved owing to advances in understanding of the disease and to the advent of novel therapeutic approaches [4]. However, because disease activity not only influences the choice of therapeutic regimens but also predicts relapse or treatment-refractoriness during follow-up, it is important to accurately determine the activity of AAV [5]. Currently, several methods to assess the disease activity of AAV have been proposed, such as Birmingham vasculitis activity score (BVAS), physician global assessment, disease extent index, and five factor score (FFS) [6–8]. Although BVAS is the most widely used index to determine AAV disease activity, the presence of observer variability and the complexity of BVAS assessment makes it difficult to apply in clinical practice, and an unmet need for a biomarker to assess disease activity of AAV has been consistently raised [7].

Chitinase-3-like 1 protein (YKL-40) is a secreted glycoprotein originally isolated from human osteosarcoma cells [9]. YKL-40 is approximately 40 kDa in size and is expressed and secreted by various cell types such as neutrophils, macrophages, vascular smooth muscle cells, chondrocytes, and synovial cells. Furthermore, it is considered to be associated with inflammation and tissue remodelling, and is increased in the sera of patients with large vessel vasculitides of giant cell arteritis and Takayasu arteritis [10–13]. In addition, previous studies suggested that serum YKL-40 is elevated in those with various medical conditions such as infections, autoimmune diseases, and cancers [14]. On the other hand, it was reported that elevated serum YKL-40 levels are associated with poor prognostic outcomes of various cancers [15] and with disease activity in rheumatoid arthritis (RA), psoriatic arthritis, and inflammatory bowel diseases [16–18]. Particularly, serum YKL-40 has been extensively studied in RA, and persistently increased serum YKL-40 was also indicative of radiologic disease progression [19]. Thus, serum YKL-40 is included in the multi-biomarker disease activity score as an objective measure to assess RA disease severity [20]. Because activated neutrophils and macrophages are important effector cells known to contribute to endothelial and organ damages in AAV and are responsible for the production of YKL-40 [1, 21], it can be speculated that serum YKL-40 may be elevated in AAV. However, so far, there has been no report on the clinical implications of serum YKL-40 in AAV patients. Hence, in this study, we investigated whether serum YKL-40 is associated with AAV disease activity.

## Methods

### Patients

We selected 60 AAV patients who were enrolled in the Severance Hospital ANCA-associated Vasculitides (SHAVE) cohort from November 2016 to June 2018. The SHAVE cohort is an observational cohort that exclusively included AAV patients (MPA, GPA, and EGPA), which was established in a major tertiary referral centre in South Korea. All patients were first classified as AAV at the Department of Rheumatology in Severance Hospital, fulfilled the 2007 European Medicines Agency algorithms, and the 2012 Revised International Chapel Hill Consensus Conference Nomenclature of Vasculitides [22, 23]. Serum samples were collected and stored at diagnosis of AAV and on every 3 month-visit day, when clinical and laboratory data were obtained. Particularly, on the visit day of blood sampling, patients with serious medical conditions other than AAV, such as cancers and infections, were excluded in this study. This study was approved by the Institutional Review Board of Severance Hospital (4-2016-0901) and the patients’ written informed consent was obtained at the time of blood sampling.

### Clinical and laboratory data and blood sampling

Clinical data comprised of AAV classification, age, gender, new onset AAV, disease duration, and clinical features. We evaluated items of both BVAS and vasculitis damage index (VDI) as clinical features. Perinuclear (P)-ANCA and cytoplasmic (C)-ANCA were detected using immunofluorescent assays, and MPO-ANCA and PR3-ANCA were measured using ELISA kits for anti-MPO and anti-PR3 (Thermo Fisher Scientific/Phadia, Freiburg, Germany). For laboratory data, the results of white blood cell, neutrophil, and platelet counts, erythrocyte sedimentation rate (ESR), C-reactive protein (CRP), creatinine, aspartate aminotransferase, and alanine aminotransferase (ALT) were collected. We also reviewed immunosuppressive drugs administered on the day of blood sampling using the Korean Drug Utilization Review system. Whole blood was obtained from each AAV patient and serum was isolated and stored at − 80 °C on the same day of collecting clinical and laboratory data.

### AAV-related parameters and definition of disease status

On the visit day of blood sampling, four categories of AAV-related parameters were assessed: BVAS (version 3) as an index for the current AAV activity and severity [7], FFS as a predictor of the prognosis of AAV [8], VDI as an organ-based damage assessment [24], and the Korean version of the short form-36 (SF-36) [25] as an index of physical and mental function. We defined patients as having severe AAV by stratifying AAV patients into three groups according to the tertile of BVAS, and the lower limit of the highest tertile was set as the cut-off for severe AAV (BVAS ≥ 12). In addition, AAV patients with FFS ≥ 2 were defined as having high FFS [8]. Patients were classified as having new onset or persistent disease when the disease duration was less than and over 1 month, respectively. The definition of active disease and remission was set based on the European League Against Rheumatism recommendations [26].

### Serum YKL-40 measurement

Serum YKL-40 was measured using the stored serum samples of 60 AAV patients. Serum samples of patients with systemic lupus erythematosus (SLE) (*n* = 30), RA (*n* = 28), osteoarthritis (OA) (*n* = 22), and healthy control (HC) (*n* = 40) were used as controls. ELISA kits for serum YKL-40 measurement were purchased from Quidel (CA, USA), and the levels of serum YKL-40 were measured according to the manufacturer’s instructions. Briefly, 20 μL of each serum sample was added to each well and incubated with 100 μL of capture solution for 60 min at room temperature. After four washes with washing buffer, 100 μL of enzyme conjugate was added to each well. Again, the plate was covered with a plate sealer and incubated for 60 min at room temperature. The plate was then washed four times with washing buffer. Next, 100 μL of substrate solution was added to each well and the plate incubated for 60 min at room temperature. Following this final period of incubation, 100 μL of stop solution (1 N sulphuric acid) was added to each well and the OD value determined at 405 nm. For external validation, the serum samples of patients (*n* = 35) that were diagnosed as AAV at Imperial College of London, the Centre for Inflammatory Diseases, were used (application number 04/Q0406/25).

### Immunohistochemical staining of kidney tissue

Immunohistochemistry was performed on formalin-fixed paraffin-embedded kidney tissue obtained from patients with non-proliferative glomerular disease (thin basement membrane, *n* = 3; disease controls) and from ANCA-associated glomerulonephritis (AAGN) (*n* = 5) provided by the Imperial College NHS Trust Tissue Bank (application number R15072), in accordance with local research ethics committee approval. Following heat-induced antigen retrieval, sections were incubated with anti-YKL-40 primary antibody (Ab77528, Abcam, Cambridge, UK) overnight at 4 °C, developed using an HRP-conjugated secondary system (Vector Labs, Peterborough, UK) according to manufacturer’s instructions, and counterstained with haematoxylin.

### Statistical analysis

Distribution of normality in continuous data was evaluated using Kolmogorov-Smirnov test, and continuous variables were presented as mean with standard deviation or median with interquartile range, as appropriate. Differences of two continuous variables were compared using the Student’s *t*-test and Mann-Whitney *U* test, whereas Kruskal-Wallis test was conducted for comparing two or more variables. Categorical variables are presented as frequencies and percentages. Correlations between laboratory variables, BVAS, FFS, and SF-36 scores were assessed using univariable linear regression analysis. Standardised correlation coefficients between laboratory data, BVAS, FFS, and SF-36 scores were calculated using multivariable linear regression analysis with a forward entry method including variables that showed statistical significance in univariable analysis. The optimal cut-off of serum YKL-40 for severe AAV and high FFS was calculated using the receiver operator characteristic (ROC) curve analysis. Calculation of the relative risk (RR) was performed using the contingency tables and the chi-square test, and a paired *t*-test was done to evaluate the changes of serum YKL-40 levels after reduction of AAV activity. Statistical analyses were performed using MedCalc statistical software version 18.6 (MedCalc Software, Ostend, Belgium) or GraphPad Prism software version 5.0 (GraphPad Software, San Diego, CA, USA). A two-tailed *p* < 0.05 was considered statistically significant.

## Results

### Baseline characteristics of patients with AAV

The baseline characteristics of AAV patients are shown in Table 1. Thirty-two patients (53.3%) were classified as MPA, 17 (28.3%) as GPA, and 11 (18.3%) as EGPA. The mean age of the patients was 59.4 and 40 (66.7%) were women. Twenty-three patients (38.3%) had new onset AAV, and the median disease duration was 2.4 months. For AAV-related parameters, the median BVAS, FFS, and VDI scores were 7.0, 1.0, and 3.0, respectively. The mean SF-36 physical component summary (PCS) and mental component summary (MCS) scores were assessed as 50.5 and 58.3, respectively. The most common clinical feature was pulmonary manifestation (61.7%) followed by renal manifestation (55.0%). MPO-ANCA (or P-ANCA) was detected in 37 patients (61.7%) and PR3-ANCA (or C-ANCA) in seven patients (11.7%). The median white blood cell and neutrophil counts, ESR, CRP, and serum YKL-40 were 7345.0/mm^3^, 5000.0/mm^3^, 30.0 mm/h, 1.4 mg/L, and 189.2 ng/mL, respectively. Concerning immunosuppressive agents currently being administered, glucocorticoids (70.0%) were the most commonly used, followed by azathioprine (23.3%), cyclophosphamide (13.3%), and tacrolimus (5.0%).

**Table 1:** Baseline characteristics of patients with AAV (*n* = 60)

	Values
**Classification,** ***n*** **(%)**	
MPA	32 (53.3)
GPA	17 (28.3)
EGPA	11 (18.3)
**Demographic data**	
Age, years	59.4 (14.3)
Female gender, *n* (%)	40 (66.7)
New onset AAV, *n* (%)	23 (38.3)
Disease duration, months^†^	2.4 (23.9)
**AAV-related parameter**	
BVAS^†^	7.0 (10.5)
FFS (2009) ^†^	1.0 (1.0)
VDI^†^	3.0 (2.0)
SF-36 PCS score	50.5 (22.4)
SF-36 MCS score	58.3 (20.1)
**Clinical features (organ involvement),** ***n*** **(%)**	
General manifestation	17 (28.3)
Cutaneous manifestation	7 (11.7)
Mucous membrane and eye manifestation	3 (5.0)
Ear, nose, and throat manifestation	25 (41.7)
Pulmonary manifestation	37 (61.7)
Cardiovascular manifestation	3 (5.0)
Abdominal manifestation	1 (1.7)
Renal manifestation	33 (55.0)
Nervous system manifestation	12 (20.0)
**ANCA positivity,** ***n*** **(%)**	
ANCA double positivity	1 (1.7)
MPO-ANCA (or P-ANCA) positivity	37 (61.7)
PR3-ANCA (or C-ANCA) positivity	7 (11.7)
ANCA negativity	17 (28.3)
**Laboratory data**	
White blood cell count, /mm^3†^	7345.0 (5380.0)
Neutrophil count, /mm^3†^	5000.0 (4790.0)
Platelet count, × 1000/mm^3†^	254.0 (139.0)
ESR, mm/h^†^	30.0 (31.5)
CRP, mg/L^†^	1.4 (8.1)
Creatinine, mg/dL^†^	1.0 (1.4)
AST, IU/L^†^	18.0 (7.0)
ALT, IU/L^†^	18.5 (13.5)
Serum YKL-40, ng/mL^†^	189.2 (190.7)
**Concurrent immunosuppressive agents,** ***n*** **(%)**	
Glucocorticoid	42 (70.0)
Cyclophosphamide	8 (13.3)
Rituximab	1 (1.7)
Azathioprine	14 (23.3)
Tacrolimus	3 (5.0)
Mycophenolate mofetil	2 (3.3)
Methotrexate	2 (3.3)

Values are expressed as mean (standard deviation) or in number (percentages)

^†^Continuous variables that were non-normally distributed are expressed as median (interquartile range)

*AAV* ANCA-associated vasculitis, *ANCA* anti-neutrophil cytoplasmic antibody, *MPA* microscopic polyangiitis, *GPA* granulomatosis with polyangiitis, *EGPA* eosinophilic granulomatosis with polyangiitis, *BVAS* Birmingham vasculitis activity score, *FFS* Five factor score, *VDI* Vasculitis damage index, *SF-36* Short form-36, *PCS* Physical component summary, *MCS* Mental component summary, *MPO* myeloperoxidase, *P* perinuclear, *PR3* proteinase 3, *C* cytoplasmic, *ESR* erythrocyte sedimentation rate, *CRP* C-reactive protein, *AST* aspartate aminotransferase, *ALT* alanine aminotransferase

### Serum YKL-40 levels according to disease activity and ANCA positivity

**Fig. 1:**
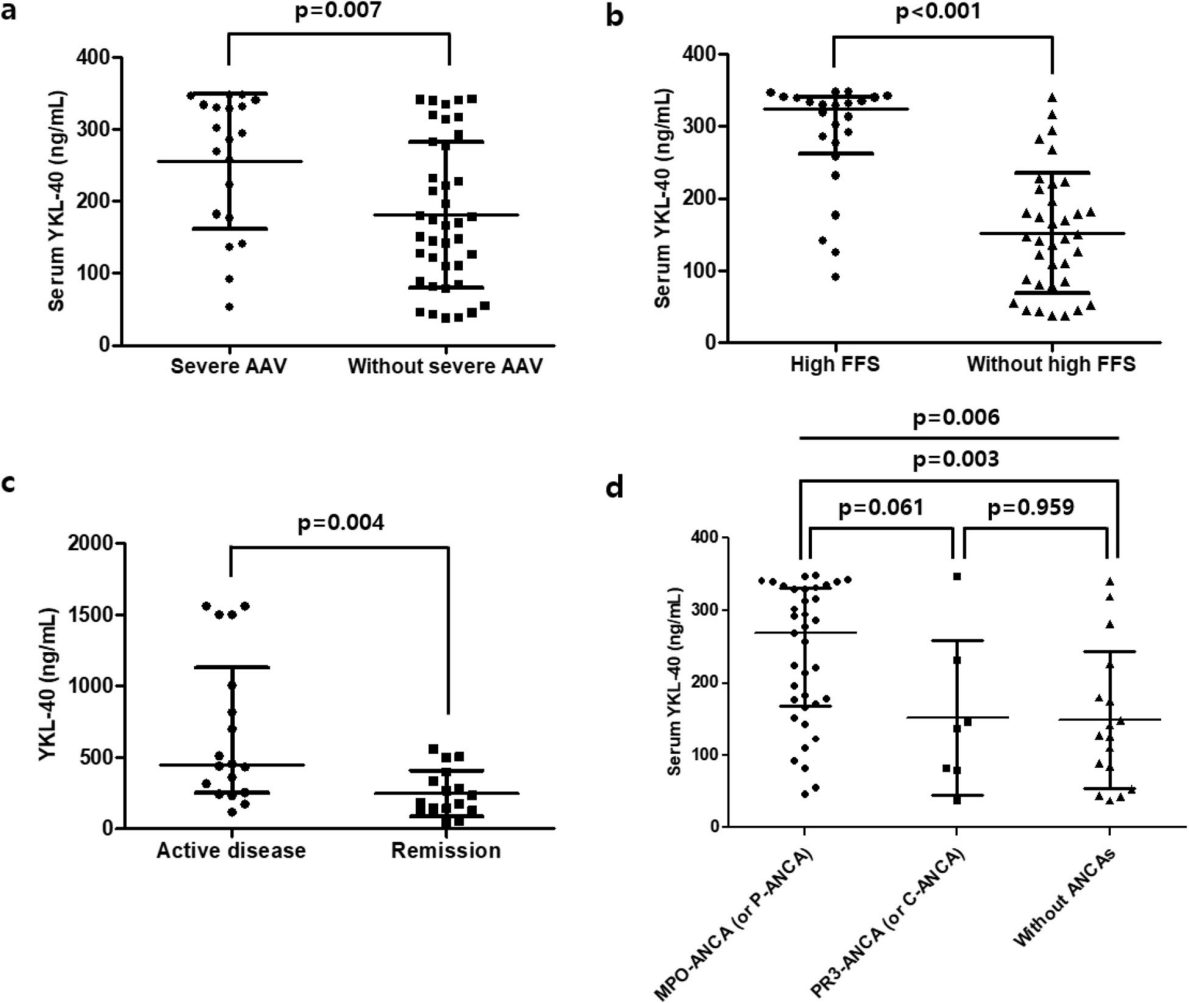
Serum YKL-40 levels in patients with severe AAV, high FFS, active disease, and ANCA positivity. Serum YKL-40 levels were significantly higher in patients with **a** severe AAV and **b** high FFS compared to those without. **c** Serum YKL-40 was higher in patients with active disease compared to those in remission from the UK samples. **d** MPO-ANCA (or P-ANCA)-positive AAV patients had significantly higher serum YKL-40 than those with ANCA negative patients. Data are shown as mean ± standard deviation or median (interquartile range), as appropriate. Differences of two continuous variables were compared using the Student’s *t*-test and Mann-Whitney *U* test, whereas Kruskal-Wallis test was conducted for comparing two or more variables. AAV, ANCA-associated vasculitis; ANCA, anti-neutrophil cytoplasmic antibody; FFS, Five factor score; MPO, myeloperoxidase; P, perinuclear; PR3, proteinase 3; C, cytoplasmic

We divided AAV patients into two subgroups based on severe AAV and high FFS, and compared serum YKL-40 levels between patients with or without severe AAV or high FFS. Patients with severe AAV exhibited higher mean serum YKL-40 levels than those without (*p* = 0.007) (Fig. 1a). In addition, serum YKL-40 levels were significantly elevated in patients with high FFS compared to those without (*p* < 0.001) (Fig. 1b). Increased levels of YKL-40 were also found in patients with active disease compared to those in remission in sera provided from an independent study population from the UK (*p* = 0.004) (Fig. 1c). Furthermore, when AAV patients were assigned to three subgroups according to ANCA positivity, serum YKL-40 levels were significantly higher in patients with MPO-ANCA (or P-ANCA) than in those without ANCAs (*p* = 0.003) (Fig. 1d).

### Linear regression analyses of laboratory variables for BVAS and FFS

In terms of the association between laboratory variables and BVAS, white blood cell count, neutrophil count, ESR, CRP, and serum YKL-40 were significantly correlated with BVAS in univariable linear regression analysis. In multivariable linear regression analysis, serum YKL-40 (standardised *β* 0.301, 95% confidence interval [CI] 0.005, 0.035, *p* = 0.012) were significantly associated with BVAS, together with CRP (standardised *β* 0.371, 95% CI 0.035, 0.153, *p* = 0.002) (Table 2). Regarding the association between laboratory variables and FFS, CRP, creatinine, ALT, and serum YKL-40 were significantly correlated with FFS in univariable linear regression analysis. However, in multivariable linear regression analysis, serum YKL-40 (standardised *β* 0.476, 95% CI 0.002, 0.006, *p* < 0.001) was significantly associated with FFS, together with creatinine (standardised *β* 0.329, 95% CI 0.055, 0.274, *p* = 0.004) (Table 2). The optimal cut-offs of serum YKL-40 were calculated as 221.3 ng/mL for severe AAV (area 0.710, 95% CI 0.578, 0.820; *p* = 0.003) and 227.1 ng/mL for high FFS (area 0.870, 95% CI 0.759, 0.943; *p* < 0.001) using ROC analysis.

**Table 2:** Linear regression analyses of laboratory variables for the current BVAS and FFS

	Univariate analysis			Multivariate analysis	
	Regression coefficient (crude B)	Correlation coefficient (*R* = *β*)	*p*-value	Standardised *β*	*p*-value
**Laboratory variables vs. BVAS**					
White blood cell count	0.001	0.271	0.037		
Neutrophil count	0.001	0.393	0.002		
Platelet count	0.013	0.192	0.141		
ESR	0.073	0.350	0.006		
CRP	0.113	0.446	< 0.001	0.371	0.002
Creatinine	0.870	0.246	0.058		
AST	− 0.025	− 0.054	0.681		
ALT	0.097	0.199	0.128		
Serum YKL-40	0.026	0.393	0.002	0.301	0.012
**Laboratory variables vs. FFS**					
White blood cell count	0.000	0.043	0.745		
Neutrophil count	0.000	0.114	0.385		
Platelet count	− 0.001	− 0.109	0.406		
ESR	0.006	0.195	0.136		
CRP	0.010	0.270	0.037		
Creatinine	0.284	0.567	< 0.001	0.329	0.004
AST	0.014	0.213	0.102		
ALT	0.018	0.263	0.043		
Serum YKL-40	0.006	0.641	< 0.001	0.476	< 0.001

*BVAS* Birmingham vasculitis activity score, *FFS* Five factor score, *ESR* Erythrocyte sedimentation rate, *CRP* C-reactive protein, *AST* aspartate aminotransferase, *ALT* alanine aminotransferase

### Linear regression analyses of laboratory variables for SF-36 scores

We next evaluated the correlation between laboratory variables and SF-36 scores. Univariable linear regression analysis revealed that white blood cell, neutrophil, platelet counts, ESR, CRP, and serum YKL-40 were significantly associated with SF-36 PCS score. In multivariable analysis, serum YKL-40 (standardised *β* − 0.390, 95% CI − 0.133, − 0.035, *p* = 0.001) and platelet count (standardised *β* − 0.325, 95% CI − 0.125, − 0.022, *p* = 0.006) were inversely associated with SF-36 PCS score. Similarly, serum YKL-40 (standardised *β* − 0.297, 95% CI − 0.105, − 0.010, *p* = 0.018), together with CRP (standardised *β* − 0.269, 95% CI − 0.385, − 0.018, *p* = 0.032), revealed to be inversely correlated with SF-36 MCS score (Additional file 1).

### Serum YKL-40 levels and AAV classification, new onset AAV, and items of BVAS

First, we compared serum YKL-40 among MPA, GPA, and EGPA patients and found that serum YKL-40 levels were significantly higher in patients with MPA and GPA than in those with EGPA (Additional file 2a). In addition, serum YKL-40 levels did not differ between patients with new onset and persistent MPA and EGPA, whereas new onset GPA patients had higher serum YKL-40 compared to those with persistent GPA (*p* = 0.019) (Additional file 2b). Regarding clinical features, serum YKL-40 levels were significantly higher in AAV patients with renal manifestation compared to those with ear, nose, throat, pulmonary, and nervous system manifestation (Additional file 2c).

### Expression of YKL-40 in kidney tissue of AAV patients

**Fig. 2:**
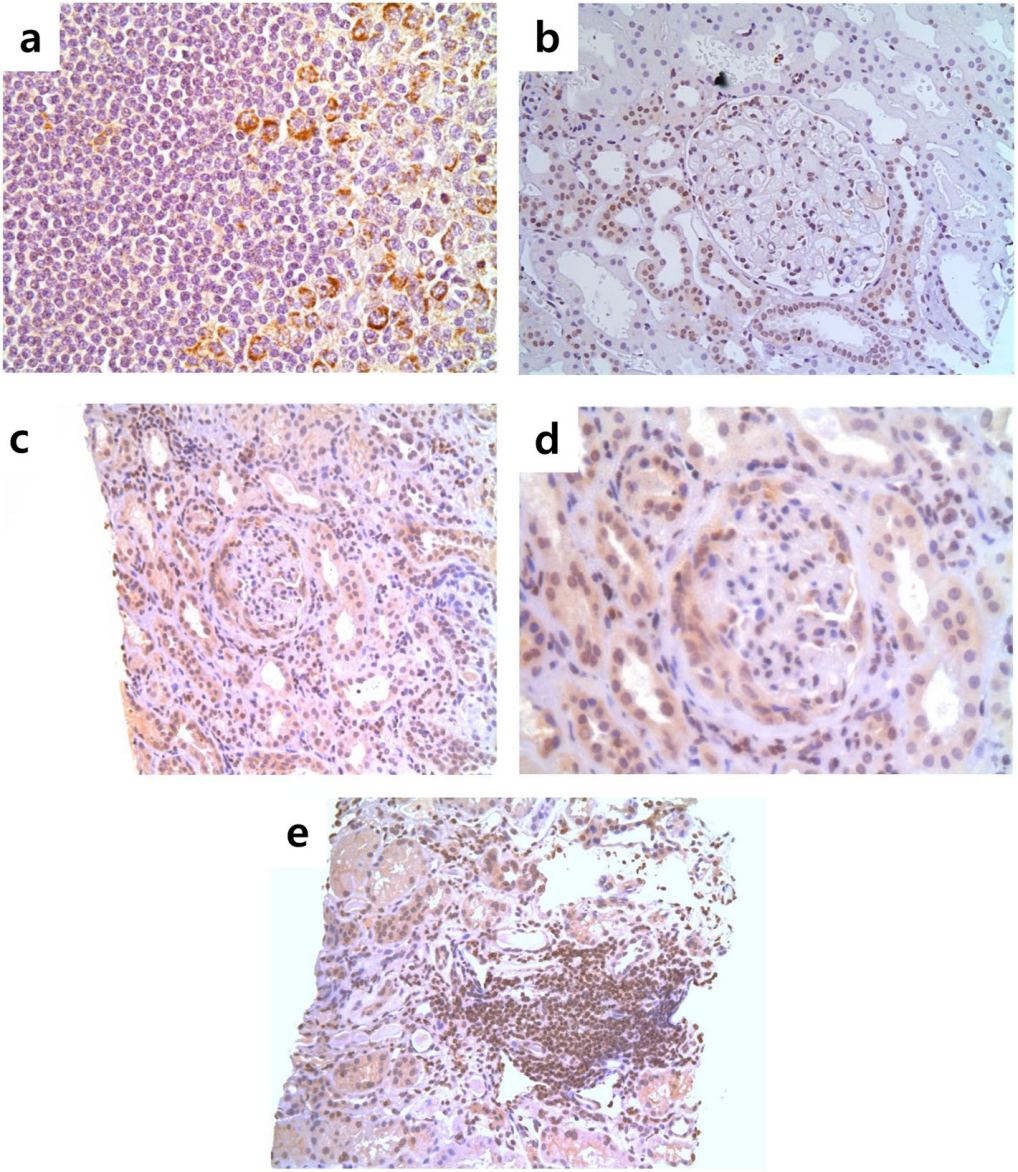
Immunohistochemistry of YKL-40 in ANCA-associated glomerulonephritis. Immunohistochemistry was performed on kidney tissues obtained from patients with non-proliferative glomerular disease (thin basement membrane, *n* = 3; disease controls) and from ANCA-associated glomerulonephritis (*n* = 5). **a** Positive tissue control stain in lymph node (× 400). **b** Negative disease control staining in non-proliferative GN (thin basement membrane lesion (× 200)). **c**, **d** YKL-40 staining in ANCA-associated glomerulonephritis, showing upregulation within tubular cells, and expression within crescentic lesions (**c** × 200; **d** × 400 same glomerulus). **e** Tubulointerstitial infiltrate in active AAV (× 200). ANCA, Anti-neutrophil cytoplasmic antibody; GN, Glomerulonephritis; AAV, ANCA-associated vasculitis

Tissue expression of YKL-40 in AAV was examined using renal biopsy material provided by the Imperial College Tissue Bank. We first validated our immunohistochemical approach in lymph nodes, which confirmed cytoplasmic expression of YKL-40 in a proportion of cells within lymphoid follicles (likely monocytes/macrophages) but not within the majority of lymphocytes (Fig. 2a). In renal tissue taken from patients with thin basement membrane disease, we found weak cytoplasmic staining for YKL-40 in some renal tubular epithelial cells, though minimal expression within the glomerular tuft, consistent with previous descriptions of YKL-40 expression in kidney tissue (Fig. 2b) [27]. In patients with AAGN, tubular expression of YKL-40 appeared to be upregulated; in addition, strong cytoplasmic staining was observed within inflammatory lesions, including areas of glomerular extra-capillary proliferation (Fig. 2c, d) and areas of tubulointerstitial inflammation (Fig. 2e).

### Comparison of serum YKL-40 levels in patients with AAV, SLE, RA, OA, and HC

**Fig. 3:**
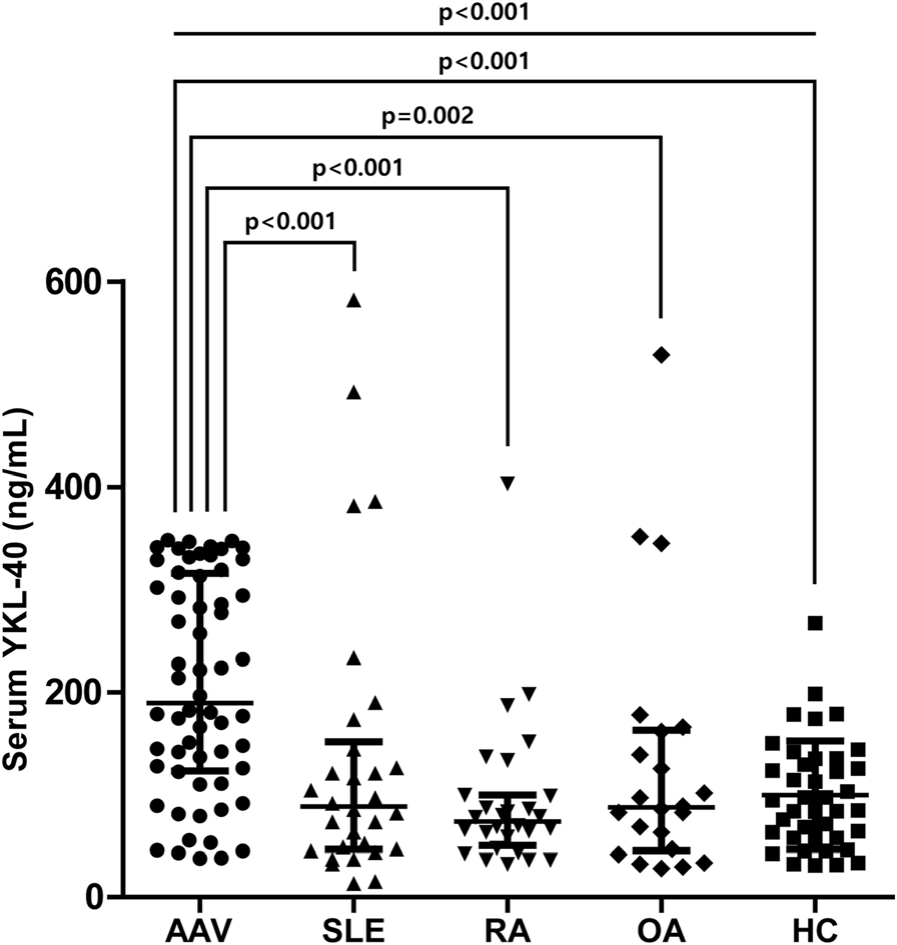
Serum YKL-40 levels in patients with AAV, SLE, RA, OA, and HC. Patients with AAV exhibited significantly higher serum YKL-40 levels compared to those with SLE, RA, OA, and HC. Data are shown as mean ± standard deviation or median (interquartile range), as appropriate. Differences of two continuous variables were compared using the Mann-Whitney *U* test, whereas Kruskal-Wallis test was conducted for comparing two or more variables. AAV, ANCA-associated vasculitis; ANCA, anti-neutrophil cytoplasmic antibody; SLE, systemic lupus erythematosus; RA, rheumatoid arthritis; OA, osteoarthritis; HC, healthy control

Because serum YKL-40 was previously reported to be elevated in other autoimmune diseases and in OA [28, 29], we compared serum YKL-40 among patients with AAV and those with SLE, RA, OA, and HC. Serum YKL-40 level was higher in patients with AAV than those with SLE, RA, and OA, as well as in those of HC (median YKL-40: AAV 189.2 ng/mL vs. SLE 88.3 ng/mL vs. RA 73.7 ng/mL vs. OA 87.4 ng/mL vs. mean YKL-40: HC 99.4 ng/mL). In contrast, no difference in serum YKL-40 was found among patients with SLE, RA, OA, and HC (Fig. 3).

### RR of severe AAV and high FFS based on serum YKL-40 levels

Patients with serum YKL-40 > 221.3 ng/mL exhibited a higher proportion of severe AAV than those without (RR 2.852, 95% CI 1.269, 6.410, *p* = 0.011) (Additional file 3a). In addition, high FFS was observed more frequently in patients with serum YKL-40 > 227.1 ng/mL than those without (RR 7.000, 95% CI 2.727, 17.972, *p* < 0.001) (Additional file 3b). On applying the cut-offs of serum YKL-40 for discriminating severe AAV and high FFS in AAV patients in the UK cohort, patients with serum YKL-40 > 221.3 ng/mL and YKL-40 > 227.1 ng/mL showed a tendency of higher risk of having severe AAV and high FFS, even though statistical significance was not found (RR 3.438, 95% CI 0.945, 12.505, *p* = 0.061 and RR 9.120, 95% CI 0.578, 143.995, *p* = 0.116) (Additional file 4a-b).

### Changes in serum YKL-40 levels after reduction of AAV activity

**Fig. 4:**
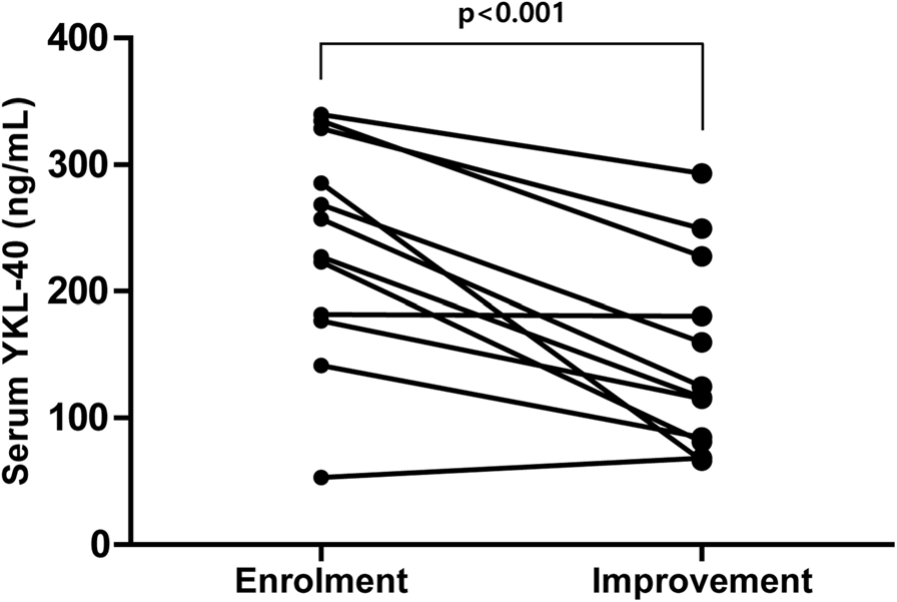
Changes in serum YKL-40 levels in patients with decreased AAV activity. In 12 patients with serial YKL-40 measurement, serum YKL-40 levels were significantly reduced with decreased BVAS at follow-up. Paired *t*-test was done to evaluate the changes of serum YKL-40 levels. AAV, ANCA-associated vasculitis; ANCA, Anti-neutrophil cytoplasmic antibody; BVAS, Birmingham vasculitis activity score

To further elucidate whether dynamic changes in serum YKL-40 could reflect changes in AAV activity, we measured serum YKL-40 levels in patients with available follow-up serum samples. Serial samples were obtainable in 12 patients exhibiting a decrease in the follow-up BVAS with an interval of 3 months or greater. Eleven of 12 patients (91.7%) exhibited a significant reduction in serum YKL-40 after improvement of AAV (*p* < 0.001) (Fig. 4).

## Discussion

In clinical practice, acute phase reactants (i.e. ESR and CRP) and ANCA titres are the most common laboratory variables to assess current AAV activity. However, owing to low specificity and reliability of these laboratory variables, a need for novel and convenient surrogate biomarkers to assess and estimate the disease activity of AAV has arisen [30, 31]. In this study, serum YKL-40 levels were significantly and independently associated with both BVAS and FFS, and the levels of YKL-40 were also higher in active disease samples provided from the UK, which strongly supports our data. In addition, the expression of YKL-40 was found to be increased in renal biopsy samples of AAGN. Furthermore, patients with serum YKL-40 levels > 221.3 ng/mL and > 227.1 ng/mL exhibited significantly higher risks of severe AAV and high FFS, respectively. It is also remarkable that serum YKL-40 levels were significantly associated with both SF-36 PCS and MCS scores, which is consistent with a previous study that demonstrated that serum YKL-40 is associated with the aggravation of functional disability in RA [32]. Finally, serial measurement of serum YKL-40 levels reflected the changes of BVAS, indicating that serum YKL-40 levels could be a potential biomarker for assessing AAV disease activity.

The significant association between serum YKL-40 levels and the disease activity of AAV may be theoretically attributable to several reasons. First, YKL-40 can be secreted by activated neutrophils as well as macrophages, which plays an important role in the pathogenesis of AAV [33]. Neutrophils, the majority of cells in innate immunity, may induce organ injury in AAV by acting both as effector cells responsible for tissue damage and by promoting chemotaxis [34]. In addition, neutrophils may contribute to the formation of neutrophil extracellular traps to perpetuate the inflammatory process in AAV [35]. On the other hand, macrophages, a likely cell type detected in AAGN biopsies, may accumulate in the target tissues and provoke organ damage in AAV at a response-to-injury phase following the resolution of acute inflammation [21]. These immunological aspects support the evidence that serum YKL-40 could be a biomarker to assess the current AAV activity. Second, serum YKL-40 can be secreted by endothelial cells as a result of vessel damage. Interestingly, two previous studies demonstrated that the levels of circulating molecules indicating vascular injury and angiogenesis are increased in AAV [36, 37]. Thus, it may be assumed that serum YKL-40 levels increase when extensive vascular damage and angiogenesis occurs. Alternatively, serum YKL-40 may also be directly associated with inflammation in AAV by increasing chemotaxis, cellular attachment, and vascular endothelial cell migration, resulting in increased vascular injury [38]. The results from a previous study have shown that neutralisation of YKL-40 inhibits angiogenesis and progression in cancer cell lines, and xenograft models support this hypothesis [39]. Third, serum YKL-40 levels may increase in AAV owing to pro-inflammatory cytokines such as interleukin (IL)-6 and IL-17. Previous studies suggested that both IL-6 and IL-17 are elevated in patients with AAV and could be important cytokines in the pathogenesis of AAV [40]. Notably, a study by Berti et al. demonstrated that blocking of IL-6 leads to clinical efficacy in AAV patients despite a relatively small number of patients [41], and another study by Gan et al., which used animal models of AAV, showed that IL-17A deficiency results in attenuation of kidney injury [42]. Because both IL-6 and IL-17A are known to enhance the expression of YKL-40 [43], it could be assumed that serum YKL-40 levels are associated with disease activity in AAV.

On evaluating the expression of YKL-40 in the kidney tissues of patients with thin basement membrane disease and AAGN, we found that the expression of YKL-40 was prominent in AAGN tissues. The upregulation of YKL-40 in the sera and kidneys in patients with AAV suggests that YKL-40 could be a biomarker to evaluate both generalised and localised inflammation in AAV. On the other hand, because it was also demonstrated that the renal expression of YKL-40 increases during ischemic renal injury [44], it is also possible that YKL-40 could be a surrogate of organ injury, and further investigations are necessary to verify the precise role of YKL-40 in AAV.

In this study, a subgroup analysis revealed that serum YKL-40 levels were significantly higher in patients with MPO-ANCA (or P-ANCA) positivity compared to those with ANCA negativity. In addition, serum YKL-40 levels were significantly higher in patients with MPA and GPA compared to EGPA, and in those with renal involvement compared to those without. Although the relations between serum YKL-40 levels and AAV variants, ANCA types, and involved organs are unclear, the presence of MPO-ANCA (or P-ANCA) could be also relevant to this observation, as a previous study discovered that sputum YKL-40 is correlated with serum YKL-40, as well as sputum MPO, vascular endothelial growth factor, IL-6, and neutrophil count in patients with asthma [45]. The results that MPO-ANCA (or P-ANCA) positivity was the most frequently detected in not only MPA patients, but also in patients with renal vasculitis related to AAV may support our assumption.

It is also noteworthy that the median serum YKL-40 levels in patients with AAV, SLE, RA, and OA were significantly lower compared to AAV patients. The serum titres of YKL-40 in RA and OA patients in this study were similar to those of previous reports [28, 46]. A significant difference in serum YKL-40 levels between AAV and other diseases might be relevant to the extensive vascular inflammation of the affected vessels in AAV, which is associated with endothelial cell injury [21]. Therefore, these findings also stress the value of determining serum YKL-40 in AAV patients. However, although serum YKL-40 level was also higher in AAV patients compared to HC, there was no difference of its level between SLE, RA, OA, and HC in our analysis. Therefore, more studies are required to validate whether serum YKL-40 may be useful in differentiating between AAV, other autoimmune diseases, and HC.

Our study has two merits. To the best of our knowledge, this is the first study to evaluate the clinical significance of serum YKL-40 levels in patients with AAV and demonstrated that it could reflect disease activity in AAV. In addition, the clinical and laboratory data in this study were derived from a monocentric prospective cohort. We could thus prevent missing data, collect cross-sectional clinical and laboratory data together with blood samples, and minimise inter-centric variation. Moreover, our results were found to be well replicated in an independent cohort. However, our study also has several limitations. First, a relatively small number of patients were included, and despite the association between serum YKL-40 levels and FFS, the prognostic implication could not be evaluated as the follow-up duration of the patients was not sufficiently long. Second, given that urinary markers such as soluble 163 and monocyte chemoattractant protein-1 have been recently proposed to be a potential biomarker in AAV, the ideal method of detecting YKL-40 (either in the blood, urine, or tissue) to provide optimal clinical information remains unclear. Third, the optimal cut-offs of serum YKL-40 that were proposed in our study could not be fully validated in an independent cohort. Fourth, we were not able to identify cell types expressing YKL-40, or determine whether YKL-40 has a functional role in AAV (regarding pathogenesis or in modulating the response to injury) in this study. Additional studies are warranted to validate our results and provide better insights concerning the role of YKL-40 in AAV.

## Conclusions

We demonstrated that serum YKL-40 levels are significantly associated with disease activity in AAV and are upregulated in kidney tissues of AAGN. In addition, serum YKL-40 levels decreased following reduction of disease activity. Therefore, our results suggest that measuring serum YKL-40 levels could provide useful information in assessing AAV disease activity.

## Supplementary Information

**Additional file 1.** Linear regression analyses of laboratory variables for the current SF-36 scores. A table presenting the association between YKL-40 and SF-36 scores.

**Additional file 2.** Serum YKL-40 levels according to AAV classification, new onset AAV, and BVAS items. (a) Serum YKL-40 was significantly higher in patients with MPA and GPA compared to those with EGPA. (b) New onset GPA patients had higher serum YKL-40 compared to those with persistent GPA. (c) Patients with renal manifestations had significantly higher serum YKL-40 compared to those with ear, nose, throat, pulmonary, and nervous system manifestations. Data are shown as mean ± standard deviation or median (interquartile range), as appropriate. Differences of two continuous variables were compared using the student’s t-test and Mann-Whitney U test, whereas Kruskal-Wallis test was conducted for comparing two or more variables. AAV, ANCA-associated vasculitis; ANCA, Anti-neutrophil cytoplasmic antibody; BVAS, Birmingham vasculitis activity score; MPA, Microscopic polyangiitis; GPA, Granulomatosis with polyangiitis; EGPA, Eosinophilic granulomatosis with polyangiitis; ENT, Ear, nose, and throat.

**Additional file 3.** Relative risk of severe AAV and high FFS based on serum YKL-40 levels. (a) Patients with serum YKL-40 levels > 221.3 ng/mL exhibited a higher proportion of severe AAV than those without. (b) High FFS was observed more frequently in patients with serum YKL-40 levels > 227.1 ng/mL than those without. Calculation of the relative risk was performed using the contingency tables and the chi-square test. AAV, ANCA-associated vasculitis; ANCA, Anti-neutrophil cytoplasmic antibody; FFS, Five factor score.

**Additional file 4 **Relative risk of severe AAV and high FFS based on serum YKL-40 levels in the UK cohort**.** (a) Patients with serum YKL-40 levels > 221.3 ng/mL tended to show a higher proportion of severe AAV than those without. (b) There was a tendency of showing higher proportion of patients with high FFS in those with serum YKL-40 levels > 227.1 ng/mL than those without. Calculation of the relative risk was performed using the contingency tables and the chi-square test. AAV, ANCA-associated vasculitis; ANCA, Anti-neutrophil cytoplasmic antibody; FFS, Five factor score; UK, United Kingdom.

## Data Availability

The datasets used and/or analysed during the current study are available from the corresponding author on reasonable request.

## Abbreviations

ALT: Alanine aminotransferase
ANCA: Anti-neutrophil cytoplasmic antibody
AAGN: ANCA-associated glomerulonephritis
AAV: ANCA-associated vasculitis
BVAS: Birmingham vasculitis activity score
C: Cytoplasmic
CRP: C-reactive protein
EGPA: Eosinophilic GPA
ESR: Erythrocyte sedimentation rate
FFS: Five factor score
GPA: Granulomatosis with polyangiitis
HC: Healthy control
IL: Interleukin
MCS: Mental component summary
MPA: Microscopic polyangiitis
MPO: Myeloperoxidase
OA: Osteoarthritis
P: Perinuclear
PCS: Physical component summary
PR3: Proteinase 3
RA: Rheumatoid arthritis
ROC: Receiver operator characteristic
RR: Relative risk
SF-36: Short form-36
SLE: Systemic lupus erythematosus
VDI: Vasculitis damage index
