# Supplementary material for: Serum chitinase-3-like 1 protein is a useful biomarker to assess disease activity in ANCA-associated vasculitis: an observational study
Source: Arthritis Res Ther. 2021 Mar 8;23:77. doi: 10.1186/s13075-021-02467-1 (PMC7938492; doi:10.1186/s13075-021-02467-1)
Supplement: Supplementary file 1 — Additional file 1. Linear regression analyses of laboratory variables for the current SF-36 scores. A table presenting the association between YKL-40 and SF-36 scores. [file 13075_2021_2467_MOESM1_ESM.docx]

**Additional file 1. Linear regression analyses of laboratory variables for the current SF-36 scores.**

|  | Univariate analysis | | |  | Multivariate analysis | |
| --- | --- | --- | --- | --- | --- | --- |
|  | Regression coefficient (Crude B) | Correlation  Coefficient (R=β) | p-value |  | Standardized β | p-value |
| **Laboratory variables vs. SF-36 PCS score** |  |  |  |  |  |  |
| White blood cell count | -0.002 | -0.282 | 0.029 |  |  |  |
| Neutrophil count | -0.002 | -0.297 | 0.021 |  |  |  |
| Platelet count | -0.075 | -0.330 | 0.010 |  | -0.325 | 0.006 |
| ESR | -0.221 | -0.321 | 0.012 |  |  |  |
| CRP | -0.327 | -0.392 | 0.002 |  |  |  |
| Creatinine | -2.147 | -0.185 | 0.157 |  |  |  |
| AST | -0.250 | -0.164 | 0.211 |  |  |  |
| ALT | -0.099 | -0.062 | 0.640 |  |  |  |
| Serum YKL-40 | -0.085 | -0.394 | 0.002 |  | -0.390 | 0.001 |
| **Laboratory variables vs. SF-36 MCS score** |  |  |  |  |  |  |
| White blood cell count | -0.001 | -0.244 | 0.060 |  |  |  |
| Neutrophil count | -0.002 | -0.267 | 0.039 |  |  |  |
| Platelet count | -0.052 | -0.255 | 0.049 |  |  |  |
| ESR | -0.201 | -0.327 | 0.011 |  |  |  |
| CRP | -0.257 | -0.342 | 0.007 |  | -0.269 | 0.032 |
| Creatinine | -2.907 | -0.279 | 0.031 |  |  |  |
| AST | -0.232 | -0.169 | 0.196 |  |  |  |
| ALT | -0.045 | -0.031 | 0.814 |  |  |  |
| Serum YKL-40 | -0.071 | -0.364 | 0.004 |  | -0.297 | 0.018 |

SF-36, Short form-36; PCS, Physical component summary; ESR, Erythrocyte sedimentation rate; CRP, C-reactive protein; AST, Aspartate aminotransferase; ALT, Alanine aminotransferase; MCS, Mental component summary.
