# Supplementary figures and images for: Serum chitinase-3-like 1 protein is a useful biomarker to assess disease activity in ANCA-associated vasculitis: an observational study
Source: Arthritis Res Ther. 2021 Mar 8;23:77. doi: 10.1186/s13075-021-02467-1 (PMC7938492; doi:10.1186/s13075-021-02467-1)

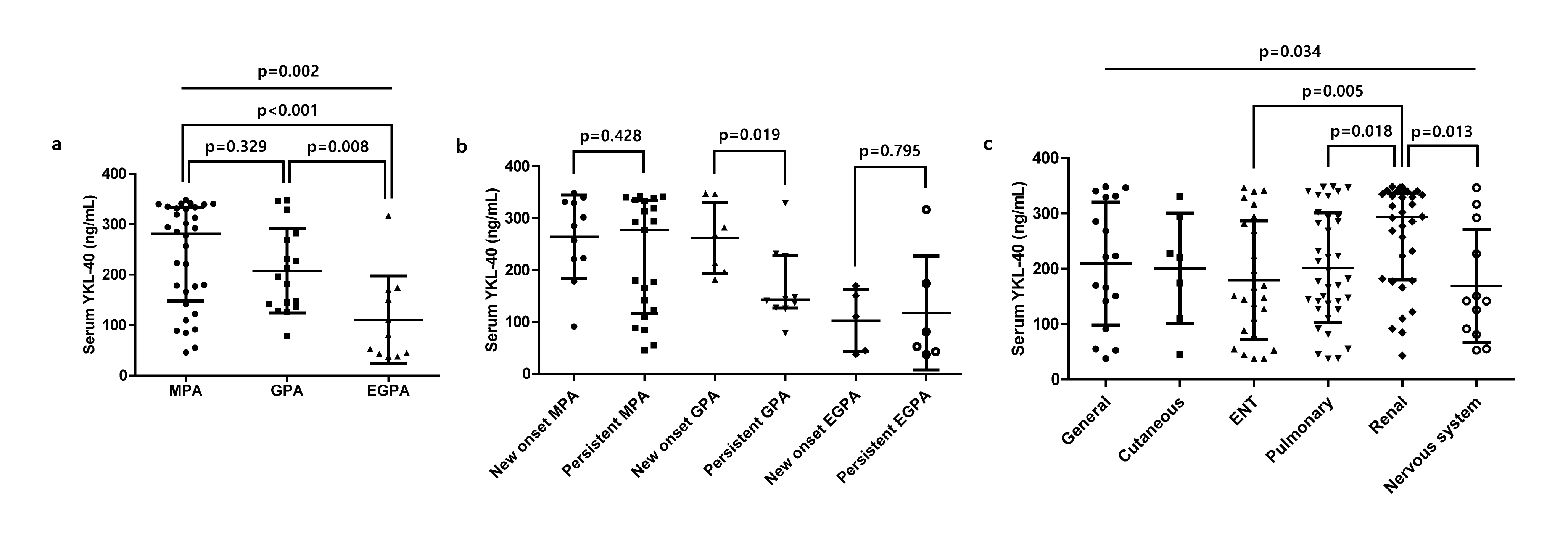

Supplement: Supplementary file 2 — Additional file 2. Serum YKL-40 levels according to AAV classification, new onset AAV, and BVAS items. (a) Serum YKL-40 was significantly higher in patients with MPA and GPA compared to those with EGPA. (b) New onset GPA patients had higher serum YKL-40 compared to those with persistent GPA. (c) Patients with renal manifestations had significantly higher serum YKL-40 compared to those with ear, nose, throat, pulmonary, and nervous system manifestations. Data are shown as mean ± standard deviation or median (interquartile range), as appropriate. Differences of two continuous variables were compared using the student’s t-test and Mann-Whitney U test, whereas Kruskal-Wallis test was conducted for comparing two or more variables. AAV, ANCA-associated vasculitis; ANCA, Anti-neutrophil cytoplasmic antibody; BVAS, Birmingham vasculitis activity score; MPA, Microscopic polyangiitis; GPA, Granulomatosis with polyangiitis; EGPA, Eosinophilic granulomatosis with polyangiitis; ENT, Ear, nose, and throat. [file 13075_2021_2467_MOESM2_ESM.tif]

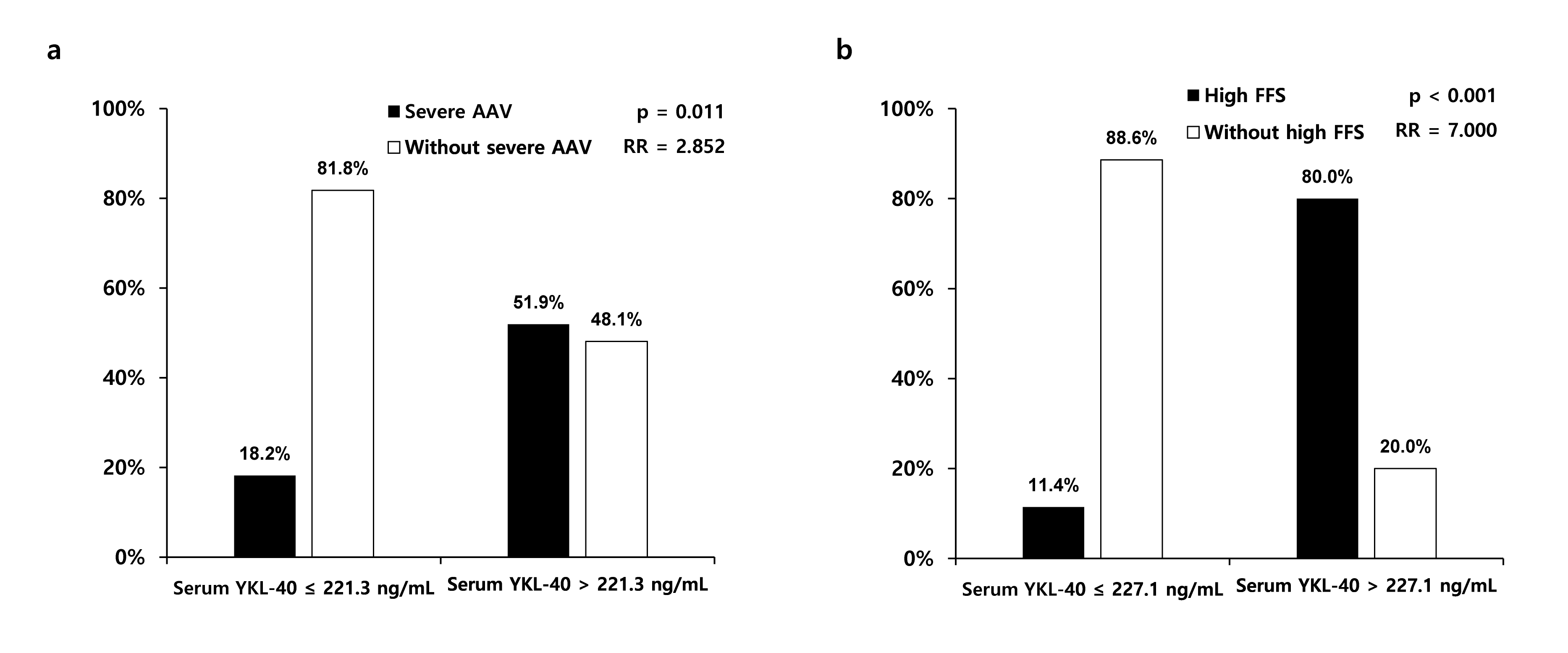

Supplement: Supplementary file 3 — Additional file 3. Relative risk of severe AAV and high FFS based on serum YKL-40 levels. (a) Patients with serum YKL-40 levels > 221.3 ng/mL exhibited a higher proportion of severe AAV than those without. (b) High FFS was observed more frequently in patients with serum YKL-40 levels > 227.1 ng/mL than those without. Calculation of the relative risk was performed using the contingency tables and the chi-square test. AAV, ANCA-associated vasculitis; ANCA, Anti-neutrophil cytoplasmic antibody; FFS, Five factor score. [file 13075_2021_2467_MOESM3_ESM.tif]

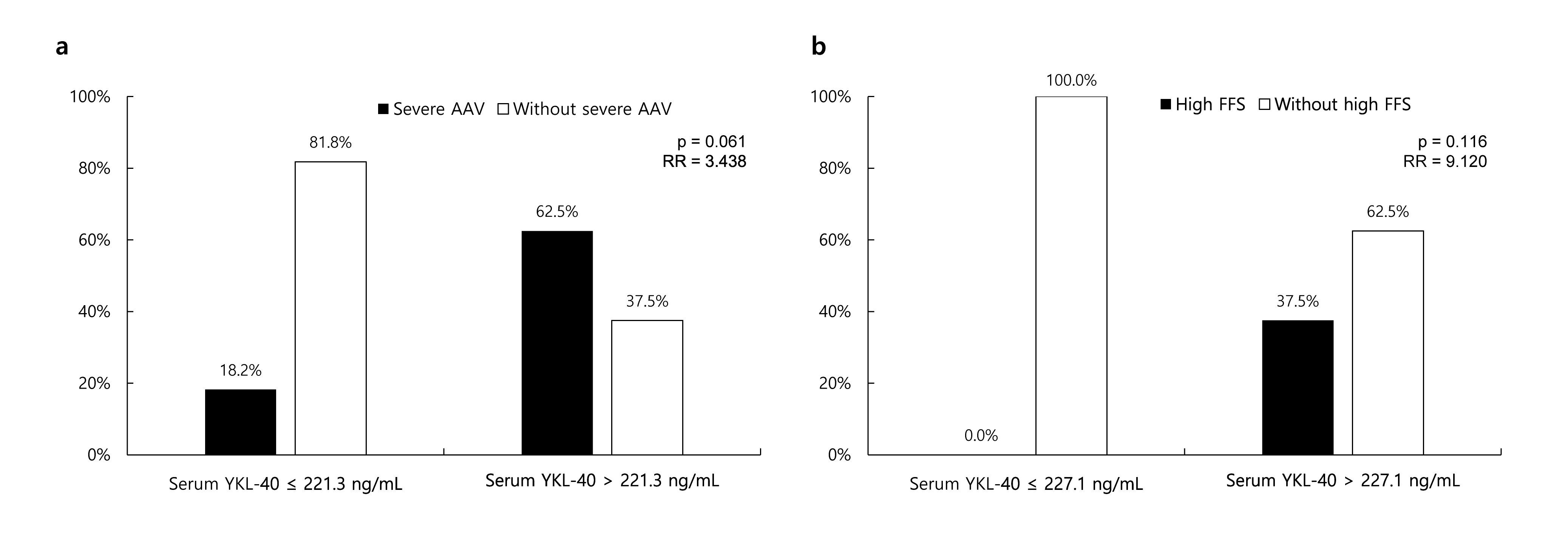

Supplement: Supplementary file 4 — Additional file 4 Relative risk of severe AAV and high FFS based on serum YKL-40 levels in the UK cohort. (a) Patients with serum YKL-40 levels > 221.3 ng/mL tended to show a higher proportion of severe AAV than those without. (b) There was a tendency of showing higher proportion of patients with high FFS in those with serum YKL-40 levels > 227.1 ng/mL than those without. Calculation of the relative risk was performed using the contingency tables and the chi-square test. AAV, ANCA-associated vasculitis; ANCA, Anti-neutrophil cytoplasmic antibody; FFS, Five factor score; UK, United Kingdom. [file 13075_2021_2467_MOESM4_ESM.tif]
